# Acute Respiratory Failure Resulting From Lambert-Eaton Myasthenic Syndrome: A Case Report and Literature Review

**DOI:** 10.7759/cureus.59516

**Published:** 2024-05-02

**Authors:** Alan R Spicer, Cleo Zarina A Reyes, Preet M Varade

**Affiliations:** 1 Neurology, University of South Florida (USF) Health, Tampa, USA; 2 Neurology, Lehigh Valley Health Network, Allentown, USA

**Keywords:** myasthenia gravis (mg), acute respiratory failure (arf), nerve conduction study (ncs), respiratory muscle weakness, lambert-eaton myasthenic syndrome

## Abstract

Lambert-Eaton myasthenic syndrome (LEMS) is a rare neuromuscular junction disorder due to auto-antibodies against presynaptic voltage-gated calcium channels (VGCC). The typical manifestation of LEMS is proximal muscle weakness, autonomic dysfunction, and areflexia; however, an atypical manifestation of LEMS is weakness of respiratory muscles, leading to acute respiratory failure. Herein, we describe a case of acute respiratory failure resulting from LEMS. Our patient was a 63-year-old woman with a past medical history of metastatic small cell lung cancer (SCLC) who presented with ambulatory dysfunction, dysarthria, and progressive dyspnea. She was intubated because of hypoxia and developed acute respiratory failure without a clear pulmonary etiology, raising the suspicion of a neuromuscular junction disorder. She was diagnosed with LEMS with a positive paraneoplastic panel for VGCC antibodies, confirmed by electromyography and nerve conduction study (EMG/NCS), and treated with intravenous immunoglobulin (IVIg). The patient's hospital stay was complicated by pneumonia, and comfort care was ultimately pursued. Our case highlights the importance of considering LEMS in patients presenting with isolated respiratory muscle weakness without focal neurological deficits. To our knowledge, this is the first report to review all reported cases of LEMS with resultant respiratory failure. We aim to establish the association of LEMS with respiratory failure so that appropriate treatment is initiated as early as possible.

## Introduction

Lambert-Eaton myasthenic syndrome (LEMS) is an autoimmune neuromuscular disorder characterized by antibodies to the P/Q-type voltage-gated calcium channels (VGCC) located on presynaptic neurons [1]. Binding to the VGCC results in decreased calcium influx into the presynaptic nerve terminal, leading to reduced presynaptic vesicular fusion and impaired release of acetylcholine into the synapse. Consequently, patients classically present with proximal muscle weakness, autonomic dysfunction, and areflexia [1]. However, an atypical presentation of LEMS is respiratory muscle weakness, which is more common in patients with myasthenia gravis (MG) [1,2].

Respiratory failure in LEMS can be precipitated after the use of drugs including calcium-channel blockers, particularly verapamil and diltiazem [3]; aminoglycosides, particularly neomycin [4]; and muscle relaxants, particularly succinylcholine [5]. Post-myocardial infarction respiratory failure in a LEMS patient has also been reported [6]. Additionally, a combination of LEMS and MG, known as overlap myasthenic syndrome, may lead to acute respiratory failure [7].

However, respiratory muscle weakness as the presenting symptom of LEMS has been relatively rarely documented, and the prognosis remains unclear. In addition to our report, only 15 cases of LEMS with respiratory failure have been reported [2-13]. We aim to illustrate the need to consider LEMS in patients with respiratory muscle weakness without focal neurological deficits.

## Case presentation

In March 2023, a 63-year-old woman presented to the emergency department as a stroke alert with acute onset of ambulatory dysfunction, acute onset of dysarthria, and progressive two-month dyspnea. Neurological examination revealed bilateral dysmetria on a finger-to-nose test, with a National Institutes of Health Stroke Scale (NIHSS) of 2. She became hypotensive (86/60 mmHg) and hypoxic (87% oxygen saturation (SpO2)) and lost consciousness. She was intubated for airway protection and admitted to the neuroscience intensive care unit (ICU) with suspicion of seizures. Her medical history included metastatic small cell lung cancer (SCLC), metastatic colon cancer in remission, and use of dexamethasone for Crohn's disease.

**Figure 1:**
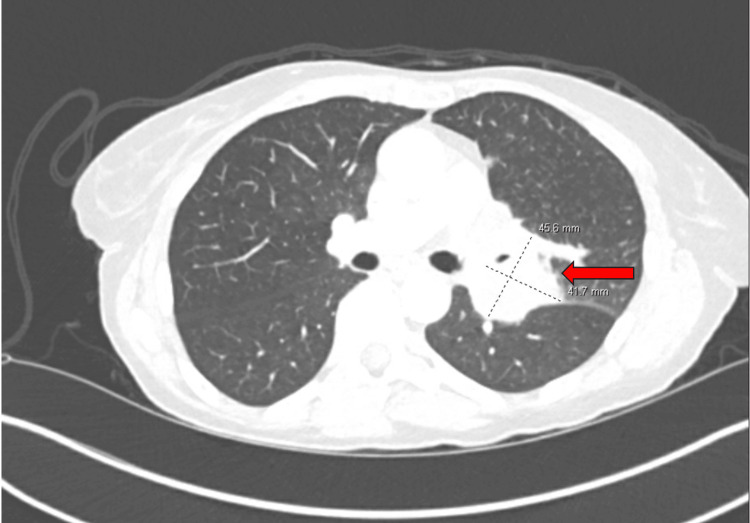
Axial CT of the chest showing left hilar mass and perihilar nodular densities from May 2022. CT: computed tomography

Regarding her cancer history, she was found to have metastatic colon cancer to both ovaries and peritoneum. She underwent chemotherapy, total abdominal hysterectomy, and bilateral salpingo-oophorectomy in 2008. Surveillance computed tomography (CT) in May 2022 revealed a new mass in her left hilum along with upper lobe perihilar nodular densities likely representing satellite metastatic lesions (Figure 1).

**Figure 2:**
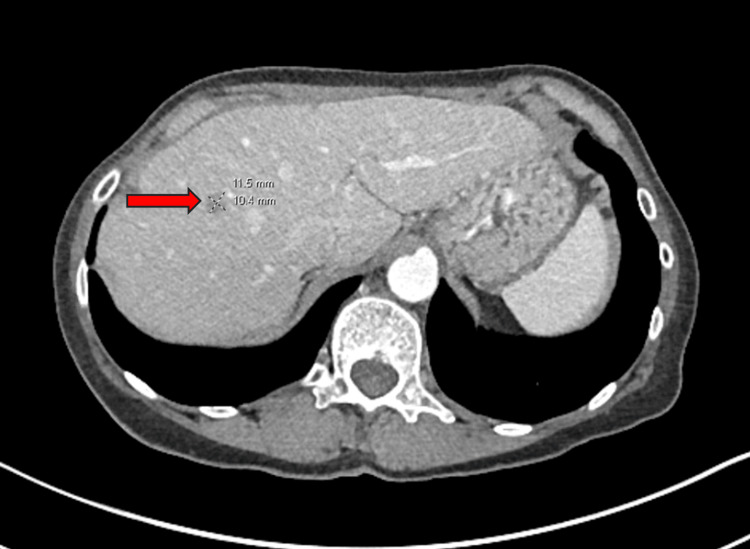
Axial CT showing low-attenuation nodule in the dome of the liver from May 2022. CT: computed tomography

The surveillance CT also showed a low-attenuation lesion involving the dome of the liver, raising suspicion of liver metastasis (Figure 2).

To conclude her encounter in May 2022, a left upper lobe endobronchial biopsy stained positive for synaptophysin and CD56 and was supportive of small cell carcinoma. She was diagnosed with SCLC with metastasis to the liver and bones and received carboplatin and etoposide. Follow-up CT in August 2022 did not show any new hypermetabolic pulmonary mass, pulmonary consolidation, or pleural effusion.

Upon her admission as a stroke alert in March 2023, a chest X-ray (CXR) showed a left basilar infiltrate. CT of the head without contrast did not show any acute large infarct, hemorrhage, or mass. Magnetic resonance imaging (MRI) of the brain with and without contrast was also unremarkable. Electroencephalography (EEG) did not show any epileptiform activity and was only notable for diffuse slowing in the theta and delta ranges, which was consistent with a diffuse encephalopathy associated with her sedation. Her lumbar puncture indicated elevated total nucleated cells but was otherwise within normal limits (Table 1).

**Table 1:** Results of lumbar puncture upon admission.

Lab	Patient	Range, units
Total nucleated cells	15	0-5, per cmm
Glucose	68	40-70, mg/dL
Protein	35	15-45, mg/dL

She continued to be lethargic but was following commands. Two weeks after admission, she was evaluated by the rapid response team for sudden unresponsiveness and agonal breathing. She was reintubated and again admitted to the ICU, where she was found to have extensor posturing of her arms and legs. On neurological examination, she did not respond to questions, had poor attention to the examiner, and did not name, repeat, or follow commands. Her brainstem reflexes were intact. She withdrew to pain in all extremities. There was an unclear etiology for her sudden unresponsiveness and posturing that presented after her arrival to the ICU.

**Figure 3:**
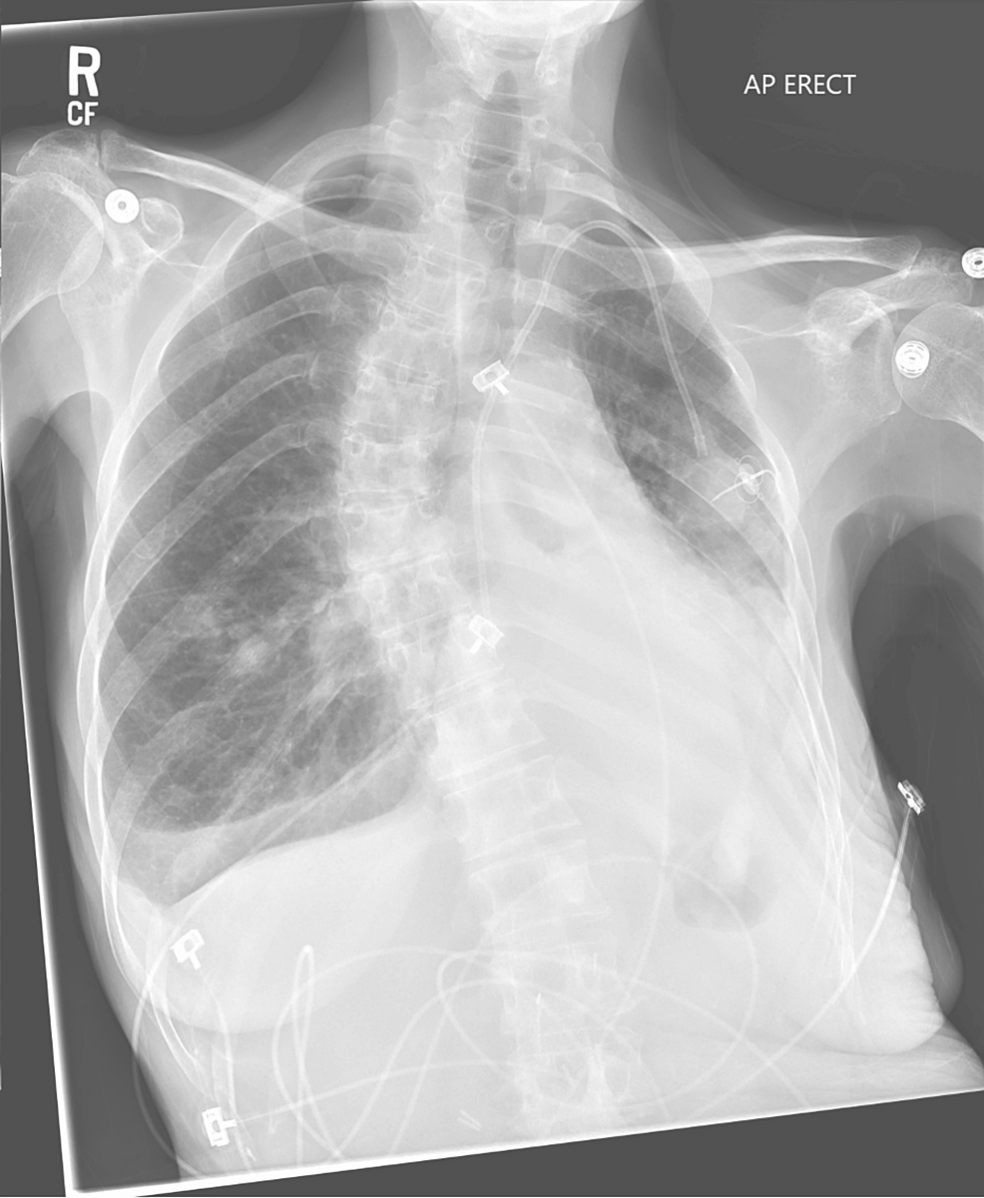
Anteroposterior CXR showing persistent left lower lobe atelectasis and right pleural effusion from March 2023. CXR: chest X-ray

Her mental status gradually improved over two days, prompting extubation; however, a pleural effusion was suspected. Thoracentesis showed 300 cc of transudative fluid with no observed improvement per imaging. She was reintubated to perform a bronchoscopy for left lung atelectasis, which showed mucous plugging of the left tracheobronchial tree. She was extubated but immediately developed respiratory failure, requiring reintubation for another 10 days. After being extubated to bilevel positive airway pressure (BiPAP), she was again reintubated for hypoxia with left lung atelectasis detected on a repeat CXR (Figure 3).

Due to the frequent episodes of respiratory distress without clear pulmonary etiology, respiratory muscular weakness from an underlying neuromuscular junction pathology was suspected. In light of the known cancer history, a paraneoplastic panel was sent that was positive for VGCC antibodies (1.29 nmol/L, reference ≤0.02 nmol/L). This raised suspicion for LEMS, so a confirmatory electromyography and nerve conduction study (EMG/NCS) was performed. Slow 3 Hz repetitive nerve stimulation of the left median nerve, recording the abductor pollicis brevis, revealed a 46% decrement at rest. There was facilitation and repair of the decrement with 10 seconds of maximum voluntary effort, with increased amplitude from 4.0 to 6.1 mV. Thus, the EMG/NCS indicated improved muscle response with repeated movements, a hallmark of LEMS [1], confirming the diagnosis. She was started on intravenous immunoglobulin (IVIg) totaling 2 g/kg over three days. After she completed IVIg therapy, she continued to have 4/5 strength in bilateral proximal upper extremities, proximal lower extremities, and distal lower extremities. 

Her clinical condition continued to deteriorate, and a CXR showed a patchy right basilar parenchymal opacity, suggesting pneumonia. She was placed on empiric vancomycin/cefepime for six days due to recurrent mucous plugging/reintubation, fever, and leukocytosis. She continued to require high-flow nasal cannula intermittently for hypoxia and was unable to wean off oxygen support. On the 28th day of admission, she remained hypoxic despite maximum oxygenation on high-flow nasal cannula and decided to pursue comfort care. She passed from a cardiopulmonary asystole four hours later. 

## Discussion

We report a case of severe respiratory muscle weakness as the manifesting symptom of LEMS. While respiratory muscle weakness has been well-documented with MG [1,2], it has been rarely observed in patients with LEMS.

Our patient initially presented as a stroke alert from ambulatory dysfunction, dysarthria, and progressive dyspnea. Emergent and limited neurological evaluation only appreciated bilateral finger-to-nose dysmetria. In retrospect, this was likely a manifestation of generalized muscular weakness that may have not been appreciated in limited neurological examination. This could further support the diagnosis of LEMS.

Of the 15 reviewed cases of LEMS with respiratory muscle weakness [2-13], ambulatory dysfunction was observed in five out of 15 cases (33%), dysarthria was observed in one out of 15 cases (7%), dyspnea was observed in four out of 15 cases (27%), and generalized weakness was observed in four out of 15 cases (27%) (Table 2).

**Table 2:** Documented cases of LEMS with respiratory failure. LEMS: Lambert-Eaton myasthenic syndrome; EMG: electromyography; VGCC: voltage-gated calcium channels; SCLC: small cell lung cancer; IVIg: intravenous immunoglobulin; y.o.: years old; M: male; F: female; 3,4-DAP: 3,4-diaminopyridine; ARDS: acute respiratory distress syndrome

Authors	Patient age/gender	Presenting symptom	EMG findings	VGCC findings	SCLC diagnosis (and timing)	Treatment	Outcome
Brehm et al. [2] (2017)	52 y.o. F	Lethargy, dyspnea	+	+	+ (during hospitalization)	Pyridostigmine, plasmapheresis, etoposide, cisplatin, chest radiation	Discharged
Gracey and Southorn [5] (1987)	59 y.o. F	Not reported	+	Not reported	+ (not reported)	Guanidine hydrochloride	Discharged
Gracey and Southorn [5] (1987)	64 y.o. M	Not reported	+	Not reported	+ (not reported)	Guanidine hydrochloride	Discharged
Gracey and Southorn [5] (1987)	66 y.o. M	Not reported	+	Not reported	+ (not reported)	Guanidine hydrochloride	Discharged
Gracey and Southorn [5] (1987)	69 y.o. M	Not reported	+	Not reported	+ (not reported)	Plasmapheresis, pancuronium bromide	Deceased from respiratory failure
Gracey and Southorn [5] (1987)	69 y.o. M	Not reported	+	Not reported	+ (not reported)	Succinylcholine	Deceased from respiratory failure
Uemura et al. [6] (2023)	58 y.o. M	Proximal muscle weakness, xerostomia	+	+	+ (during hospitalization)	Plasmapheresis, IVIg, steroid pulse therapy	Discharged
Roohi et al. [7] (2006)	70 y.o. F	Lethargy, progressive dyspnea	-	+	-	Pyridostigmine, prednisone	Discharged
Ten Brinck et al. [8] (2023)	62 y.o. F	Progressive dyspnea	initially -, then +	+	+ (during hospitalization)	3,4-DAP, IVIg, plasmapheresis, prednisone	Deceased post-extubation
Behari et al. [9] (2017)	64 y.o. M	Dysphagia, ambulatory dysfunction	+	+	+ (during hospitalization)	IVIg, carboplatin, etoposide	Discharged
Nicolle et al. [10] (1996)	73 y.o. F	Ambulatory dysfunction, generalized weakness	+	+	-	Pyridostigmine, plasmapheresis, 3,4-DAP, prednisone	Discharged
Nicolle et al. [10] (1996)	74 y.o. M	Ambulatory dysfunction, generalized weakness	+	Not reported	+ (during hospitalization)	Plasmapheresis, 3,4-DAP, chemotherapy, prednisone	Discharged
Barr et al. [11] (1993)	69 y.o. F	Generalized weakness, dyspnea, ambulatory dysfunction	+	Not reported	-	Prednisone, plasmapheresis, IVIg, guanidine hydrochloride, pyridostigmine	Deceased from ARDS
Yamada et al. [12] (1990)	63 y.o. M	Ptosis, diplopia	+	Not reported	+ (postmortem)	Plasmapheresis, anticholinesterase, guanidine hydrochloride, corticosteroid	Deceased from pneumonia
Smith and Wald [13] (1996)	62 y.o. F	Progressive weakness, ambulatory dysfunction, dysarthria	+	Not reported	+ (during hospitalization)	Pyridostigmine, plasmapheresis, 3,4-DAP, prednisone	Discharged

Ten out of 15 cases (66%) were diagnosed with SCLC during hospitalization, while one additional case was diagnosed with SCLC postmortem (Table 2). This is consistent with the established clinical association of SCLC and LEMS [1]. Notably, one case did not diagnose SCLC; however, there was a lesion "highly suspicious" of SCLC on imaging, but the patient refused a biopsy [8]. Our patient was unique in that their SCLC had responded to chemotherapy and did not show pulmonary mass, consolidation, or pleural effusion on a surveillance CT done 10 months prior to admission.

Fourteen out of 15 cases (93%) displayed improved muscle response with repeated stimulation on EMG, a characteristic finding of LEMS [1]. The only patient without characteristic EMG findings was the overlap myasthenic syndrome case [7], which may be attributed to the mutually exclusive findings of LEMS and MG on EMG.

The diagnosis of LEMS is based on clinical features; EMG findings and detection of specific VGCC antibodies are utilized to confirm the diagnosis [1]. In all reported cases, a clear pulmonary etiology of respiratory muscle weakness was not identified, leading to serologic testing and/or EMG [2-13], similar to our patient, whose LEMS diagnosis was confirmed by VGCC antibodies and EMG testing. The electrophysiologic triad was first reported by Eaton and Lambert, which includes a low compound muscle action potential (CMAP) amplitude at rest, a decremental response at low rates, and an incremental response at high-rate stimulation or brief exercise [14]. Additionally, P/Q-type VGCC antibodies have been observed in approximately 80-90% of patients with LEMS [1,15]. In patients with SCLC and LEMS, antibodies against SOX1 have also been detected [16].

The treatment of LEMS includes the management of underlying malignancy and symptomatic treatment. The first-line treatment for LEMS is considered 3,4-diaminopyridine (3,4-DAP) [1], which works by blocking calcium-dependent potassium channels, thereby prolonging the action potential at the motor nerve terminal. This leads to an increase in presynaptic influx of calcium and thereby enhancement of acetylcholine release, which facilitates improved muscle function [1].

For rapidly progressive LEMS or for refractory cases, IVIg has been the primary treatment with immunosuppressive agents and plasma exchange as other viable options [1]. In the most recent cases documented (within the last six years), patients received either plasma exchange, IVIg, chemotherapy, or a combination of the three treatments (Table 2). The efficacy of these treatments and prognosis of these cases each remain unclear and limited by the small sample size of reported cases. Of the reviewed literature, five out of 15 cases (33%) expired during hospitalization (Table 2). Pyridostigmine was used in five cases, and four out of five cases (80%) were successfully discharged, although one of these four required readmission and subsequent discontinuation of pyridostigmine due to *Clostridium difficile* infection [7]. Our patient did not improve clinically despite IVIg therapy, potentially related to her prolonged hospitalization, delay in diagnosis, and other medical complications. Of the other cases treated with IVIg, two out of four cases (50%) were discharged (Table 2).

## Conclusions

To our knowledge, this report is the first to review all cases of LEMS with resultant respiratory failure. We highlight the importance of considering neuromuscular junction disorders, particularly LEMS, in patients presenting with isolated profound respiratory muscle weakness without focal neurological deficits. Establishing the association of LEMS with respiratory disease could lead to timely identification of LEMS, diagnosis of an underlying malignancy (if unknown), and initiation of appropriate symptomatic treatment.
